# Early life exposures contributing to accelerated lung function decline in adulthood – a follow-up study of 11,000 adults from the general population

**DOI:** 10.1016/j.eclinm.2023.102339

**Published:** 2023-12-08

**Authors:** Jorunn Kirkeleit, Trond Riise, Mathias Wielscher, Simone Accordini, Anne-Elie Carsin, Julie Dratva, Karl A. Franklin, Judith Garcia-Aymerich, Deborah Jarvis, Benedicte Leynaert, Caroline J. Lodge, Francisco Gomez Real, Vivi Schlünssen, Angelo Guido Corsico, Joachim Heinrich, Matthias Holm, Christer Janson, Bryndis Benediktsdóttir, Rain Jogi, Shyamali C. Dharmage, Marjo-Riitta Järvelin, Cecilie Svanes

**Affiliations:** a Department of Occupational Medicine, Haukeland University Hospital, Bergen, Norway; b Centre for International Health, Department of Global Public Health and Primary Care, University of Bergen, Bergen, Norway; c Department of Occupational Medicine and Epidemiology, National Institute of Occupational Health, Oslo, Norway; d Department of Global Public Health and Primary Care, University of Bergen, Bergen, Norway; e Department of Epidemiology and Biostatistics, School of Public Health, Imperial College London, London, UK; f Department of Dermatology, Medical University of Vienna, Vienna, Austria; g Unit of Epidemiology and Medical Statistics, Department of Diagnostics and Public Health, University of Verona, Verona, Italy; h ISGlobal, Barcelona, Spain; i Universitat Pompeu Fabra (UPF), Barcelona, Spain; j CIBER Epidemiología y Salud Pública (CIBERESP), Barcelona, Spain; k Institute of Health Sciences, School of Health Professions, Zürich University of Applied Sciences, Winterthur, Switzerland; l Medical Faculty, University of Basel, Basel, Switzerland; m Surgical and Perioperative Sciences, Surgery, Umeå University, Umeå, Sweden; n National Heart & Lung Institute, Imperial College, London, UK; o MRC-PHE Centre for Environment and Health, Imperial College, London, UK; p Université Paris-Saclay, UVSQ, Univ. Paris-Sud, Inserm, Équipe d'Épidémiologie Respiratoire Intégrative, CESP, Villejuif, France; q Allergy and Lung Health Unit, School of Population and Global Health, University of Melbourne, Melbourne, Victoria, Australia; r Department of Gynecology and Obstetrics, Haukeland University Hospital, Bergen, Norway; s Department of Clinical Science, University of Bergen, Bergen, Norway; t Department of Public Health, Research Unit for Environment, Occupation and Health, Danish Ramazzini Centre, Aarhus University, Aarhus, Denmark; u The National Research Center for the Working Environment, Copenhagen, Denmark; v Department of Medical Sciences and Infectious Diseases, Unit of Respiratory Diseases, IRCCS Policlinico San Matteo Foundation, Pavia, Italy; x Department of Internal Medicine and Therapeutics, University of Pavia, Pavia, Italy; y Institute and Clinic for Occupational, Social and Environmental Medicine, University Hospital, LMU Munich, Comprehensive Pneumology Center Munich (CPC-M), German Center for Lung Research (DZL), Germany; z Occupational and Environmental Medicine, Institute of Medicine, School of Public Health and Community Medicine, Sahlgrenska Academy, University of Gothenburg, Gothenburg, Sweden; aa Department of Medical Sciences, Respiratory, Allergy and Sleep Research, Uppsala University, Uppsala, Sweden; ab Medical Faculty, University of Iceland, Reykjavik, Iceland; ac Lung Clinic, Tartu University Hospital, Tartu, Estonia; ad Center for Life Course Health Research, Faculty of Medicine, University of Oulu, Oulun yliopisto, Finland; ae Unit of Primary Care, Oulu University Hospital, Oulu, Finland; af Department of Life Sciences, College of Health and Life Sciences, Brunel University London, Middlesex, UK

**Keywords:** Lung function, FVC, FEV_1_, FEV_1_/FVC ratio, Accelerated decline, Early life risk factors, Maternal smoking, Maternal asthma, Paternal asthma

## Abstract

**Background:**

We aimed to assess whether exposure to risk factors in early life from conception to puberty continue to contribute to lung function decline later in life by using a pooled cohort comprising approx. 11,000 adults followed for more than 20 years and with up to three lung function measurements.

**Methods:**

Participants (20–68 years) in the ECRHS and NFBC1966 cohort studies followed in the periods 1991–2013 and 1997–2013, respectively, were included. Mean annual decline in maximum forced expired volume in 1 s (FEV_1_) and forced vital capacity (FVC) were main outcomes. Associations between early life risk factors and change in lung function were estimated using mixed effects linear models adjusted for sex, age, FEV_1_, FVC and height at baseline, accounting for personal smoking.

**Findings:**

Decline in lung function was accelerated in participants with mothers that smoked during pregnancy (FEV_1_ 2.3 ml/year; 95% CI: 0.7, 3.8) (FVC 2.2 ml/year; 0.2, 4.2), with asthmatic mothers (FEV_1_ 2.6 ml/year; 0.9, 4.4) (FEV_1_/FVC 0.04 per year; 0.04, 0.7) and asthmatic fathers (FVC 2.7 ml/year; 0.5, 5.0), and in women with early menarche (FVC 2.4 ml/year; 0.4, 4.4). Personal smoking of 10 pack-years contributed to a decline of 2.1 ml/year for FEV_1_ (1.8, 2.4) and 1.7 ml/year for FVC (1.3, 2.1). Severe respiratory infections in early childhood were associated with accelerated decline among ever-smokers. No effect-modification by personal smoking, asthma symptoms, sex or cohort was found.

**Interpretation:**

Mothers’ smoking during pregnancy, parental asthma and early menarche may contribute to a decline of FEV_1_ and FVC later in life comparable to smoking 10 pack-years.

**Funding:**

10.13039/501100007601European Union's Horizon 2020; 10.13039/501100005416Research Council of Norway; 10.13039/501100002341Academy of Finland; University Hospital Oulu; 10.13039/501100008530European Regional Development Fund; 10.13039/501100004837Spanish Ministry of Science and Innovation; 10.13039/501100002809Generalitat de Catalunya.

**Research in context**

**Evidence before this study**

Longitudinal cohorts with data on lung function into adult life, that also include information on exposure to risk factors in the pre- and post-natal period, have reported that early life risk factors contribute to an accelerated decline in the lung function measure FEV_1_ in adult life. The most important early life risk factor found was maternal smoking during pregnancy. Although FEV_1_, FVC and FEV_1_/FVC ratio may reflect different aspects of lung physiology, different phenotypes, and potentially etiology, studies on the relationship between exposure to these early risk factors and accelerated decline in FVC are scarce.

A literature search was done in PubMed for articles published from any date to 03.10.2023, with the terms “early life”, “early life risk factors”, “lung function decline” and “accelerated lung function decline” with and without the exposure factors previously reported in the scientific literature. Additional relevant articles were identified through searching the reference lists. Only full articles published in English were used.

**Added value of this study**

A higher risk of accelerated decline of FVC was observed among participants exposed to maternal smoking during pregnancy, having a father with asthma, and early age at menarche. These findings provide evidence that childhood represents an important period of growth of lung volumes that predicts accelerated decline in FVC later in life. The association between early life risk factors and accelerated decline in both FVC and FEV_1_ was considerable and comparable to smoking 10 pack-years.

**Implications of all the available evidence**

The identified associations between early life risk factors and accelerated lung function decline might allow tailored measures for prevention. This is in particularly relevant for risk factors that can be prevented such as maternal smoking. Proper treatment of respiratory infections in early childhood might limit irreversible damage and remodeling of the lung tissue.

## Introduction

Lung function reaches its’ maximum around an age of 25 years and declines after plateauing.^1^ Accelerated lung function decline causes low forced expiratory volume in 1 s (FEV_1_) and forced vital capacity (FVC) later in life. People with low lung function more often suffer from non-communicable diseases, including chronic respiratory diseases, and die earlier.^2^, ^3^, ^4^, ^5^ Hence, identification of risk factors accelerating lung function decline allowing for tailored measures for prevention may have wide public implications.

Smoking is the strongest known risk factor for accelerated lung function decline, which in turn leads to chronic obstructive pulmonary disease (COPD).^6^ Exposure to risk factors in early life are also suggested to accelerate decline in FEV_1_ later in life, including parental smoking, parental asthma, respiratory symptoms and infections in childhood, early age at menarche, and being born at wintertime.^7^, ^8^, ^9^, ^10^, ^11^, ^12^, ^13^, ^14^, ^15^ All three major measures of spirometry (FEV_1_, FVC, and FEV_1_/FVC ratio) reflect to some extent different aspects of lung physiology, different phenotypes, and potentially etiology, and are all key parameters in clinical practice for diagnosing various obstructive and restrictive lung diseases. Although there is an increasing interest in restrictive spirometry patterns as reflected by low FVC with preserved FEV_1_/FVC given its’ link to both chronic respiratory diseases and cardiometabolic diseases,^16^ studies on the relationship between exposure to risk factors in early life and FVC decline are scarce and needed to advance this field.

In a large study population followed over more than twenty years and into their 7th decade we aimed to investigate whether exposures in early life contribute to an accelerated decline in adult lung function measured as both FEV_1_, FVC and FEV_1_/FVC. We used a pooled prospective population-based cohort comprising more than 11,000 adult participants from the European Community Respiratory Health Survey (ECRHS) and The Northern Finland Birth Cohort 1966 (NFBC1966) covering a mean age between follow-ups of 34–54 and 31–47 years, respectively.

## Methods

### Study population

European Community Respiratory Health Survey (ECRHS) is a population-based multicenter cohort (www.ecrhs.org) comprising participants (men and women aged 20–44 years at baseline) selected at random from available population-based registries in 22 countries. The participants were invited through a postal screening questionnaire in 1991–93 (ECRHS1),^17^ and reinvestigated in 1998–2002 (ECRHS2, ages 29–55) and 2010–13 (ECRHS3, ages 46–68) (Figure S1). Each survey included spirometry (Table 1 and Supplementary Material Table S1) and standardized interviewer-led questionnaires on prenatal and perinatal environment, education, lifestyle, and health (Table 1).

**Table 1:** Characteristics of the study population stratified on the two cohorts ECHRS and NFBC1966.

Characteristics	ECRHS1 (n = 5767)	NFBC1966 I (n = 5917)
Sex assigned at birth, n (%)		
Male	2769 (47.9)	2835 (47.9)
Female	3009 (52.1)	3084 (52.1)
Age, years (mean (SD))		
Baseline	34.0 (7.1)	31.2 (0.36)
First follow-up	43.1 (7.1)	46.6 (0.57)
Second follow-up	54.0 (7.1)	NA
Body mass index (BMI), kg/m^3^ (mean (SD))	23.6 (3.59)	24.4 (4.0)
FEV_1_, liters (mean, SD)	3.77 (0.81)	3.95 (0.79)
FEV_1_% of predicted (mean % (SD))^a^	1.0 (0.13)	NA
Annual decline FEV_1_, ml/year (mean (SD))	35.7 (29.2)	32.9 (22.4)
FVC, liters (mean, SD)	4.56 (1.01)	4.72 (0.99)
FVC % of predicted (mean % (SD))^a^	1.0 (0.12)	NA
Annual decline FVC, ml/year (mean (SD))	27.6 (35.9)	16.9 (26.8)
Smoking status at baseline, n (%)		
Lifetime non-smoker	2461 (44.1)	2856 (49.0)
Ex-smoker	1225 (22.0)	1256 (21.6)
Current smoker	1891 (33.9)	1716 (29.4)
Missing	201	91
Age at starting smoking, years (SD)	17.1 (3.2)	16.1 (3.0)
Asthma symptoms (≥3 respiratory symptoms, 12 months), n (%)		
No	5397 (93.4)	NA
Yes	381 (6.6)	
Missing	0	
**Early life risk factors**		
Mothers age at birth, years (mean (SD))	28.8 (6.2)	28.2 (6.7)
Mothers age at birth, n (% of cohort):		
Age ≤19 years	184 (3.9)	367 (6.2)
Age 20 through 24 years	1084 (23.1)	1639 (27.8)
Age 25 through 29 years	1380 (29.4)	1643 (27.9)
Age 30 through 34 years	1217 (25.9)	1060 (18.0)
Age 35 through 39 years	567 (12.1)	774 (13.1)
Age ≥ 40 years	268 (5.7)	405 (6.9)
Missing	1078	31
Age at menarche, females, years (SD)	12.9 (1.5)	12.9 (1.3)
Age at menarche		
Early (<12 years)	315 (15.4)	371 (12.2)
Normal (12–14 years)	1471 (72.0)	2325 (76.4)
Late (>14 years)	257 (12.6)	347 (11.4)
Missing	966	41
Mother smoked during pregnancy, n (%)		
No	4703 (81.4)	5020 (84.8)
Yes	446 (7.7)	739 (12.5)
Don't know/missing	629 (10.9)	160 (2.7)
Father smoked during childhood, n (%)		
No	1998 (34.6)	NA
Yes	3587 (62.1)	
Don't know	188 (3.3)	
Missing	5	
Caesarean section, n (%)		
No	5393 (95.5)	NA
Yes	144 (2.6)	
Don't know	110 (1.9)	
Missing	131	
Season of birth, n (%)		
Winter (Nov, Dec, Jan)	1361 (23.6)	1359 (23.0)
Others	4417 (76.4)	4560 (77.0)
Missing	0	0
Mother having asthma, n (%)		
No	5267 (91.2)	4852 (82.0)
Yes	322 (5.6)	590 (10.0)
Don't know	189 (3.3)	477 (8.1)
Missing	0	0
Father having asthma, n (%)		
No	5109 (88.4)	4904 (82.9)
Yes	332 (5.7)	465 (7.9)
Don't know	337 (5.8)	550 (9.3)
Missing	0	0
Serious respiratory infections within 5 years of age, n (%)		
No	4904 (85.4)	NA
Yes	551 (9.6)	
Don't know	323 (5.0)	
Missing	32	
Serious respiratory infection within first year of life, n (%)		
No		5721 (96.7)
Yes		198 (3.3)
Missing	NA	0
Mother's education level, n (%)		
Minimum school leaving age	3411 (64.6)	3883 (66.8)
Secondary school past minimum age	1189 (22.5)	1736 (29.8)
College or university	598 (11.3)	198 (3.4)
Don't know	83 (1.6)	–
Missing	497	102
Father's education level, n (%)		
Minimum school leaving age	2797 (53.3)	NA
Secondary school past minimum age	1304 (24.9)	
College or university	1021 (19.5)	
Don't know	125 (2.4)	
Missing	531	

The percentages are calculated based on responders (missing data is not included in the percentages).

a Based on the GLI predicted values for FEV_1_ value (3.77 L, SD 0.70) and FVC (4.57 L, SD 0.89).

The Northern Finland Birth Cohort 1966 (NFBC1966) is a follow-up study of children from the two northernmost provinces of Finland (www.oulu.fi/nfbc/).^18^^,^^19^ Ninety-six percent of women with expected delivery in 1966 were recruited through maternity health centers (12,058 live births). Offspring of participating mothers still living in northern Finland or in the Helsinki area were invited for the first and second follow-ups at the ages of 31 years (1997) and 45–47 years (2012–13), respectively (Figure S1). Clinical examination included spirometry (Table 1 and Table S1) and postal questionnaires including questions on education, lifestyle, and health (Table 1). Mothers reported on offspring prenatal and perinatal environment via questionnaires during clinical visits whilst being pregnant (1965–66).

Written informed consent was obtained from each participant prior to each follow-up. Ethical approvals were obtained from the appropriate institutional or regional ethics committees for each center (ECRHS), and from the University of Oulu Ethics Committee and the Ethical Committee of the Northern Ostrobothnia Hospital District (NFBC1966).

### Lung function measures

Pre-bronchodilation spirometry was performed by trained personnel in line with the ATS/ERS standards.^20^ The participants had their lung function followed up to three times in ECRHS and twice in NFBC1966. Only participants with valid spirometry at two or more follow-ups were included (Table S1 and Figure S1). For spirometers used, see Table S1. Main outcomes were mean annual decline in maximum FEV_1_ and in maximum FVC (pre-bronchodilator, in milliliter (ml)).

### Early life risk factors

Early life risk factors were mainly limited to the perinatal and post-natal period, and for females up to menarche. We included in the analysis early life risk factors previously reported to be either associated with accelerated lung function decline in adult life^8^^,^^9^^,^^11^^,^^13^ or lower attained lung function (Table 1).^8^, ^9^, ^10^^,^^13^^,^^14^^,^^21^, ^22^, ^23^, ^24^

Maternal age at delivery was categorized as ≤19, 20–24, 25–29, 30–34, 35–39, and ≥40 years.^24^ Maternal smoking was defined as ever/never smoking in pregnancy. Mother's smoking was in ECRHS reported by participants on behalf of their mothers by answering the question “*Did your mother ever smoke regularly during your childhood, or before you were born?*”, and if yes, the participant was asked whether the mother: 1) *stopped smoking before pregnancy*, 2) *cut down or stopped during pregnancy*, or 3) *smoked as usual during pregnancy*. In NFBC1966 the mother herself reported on whether she; 1) *never smoked/not for* 12 months *before birth*, 2) *gave up smoking before pregnancy*, or 3) *smoked in pregnancy*. In order to increase the specificity for the *in utero* exposure and to harmonize the variable to NFBC1966, the maternal smoking in ECRHS was restricted to answering positive to the question on whether the mother smoked as usual during pregnancy. Father's smoking during childhood (ECRHS) was defined according to the question: *“Did your father ever smoke regularly during your childhood?*”. Having been delivered by Caesarean section (ECRHS) was defined by “*Were you delivered by Caesarean section*”. Season of birth was defined as born during winter (November, December and January) or not.^11^ Parental history of asthma were prospectively reported by parents themselves in NFBC1966 and retrospectively recalled by participants in ECRHS (*“Did your mother/father ever have asthma?*”). Parental age at completed full time education was categorized as: 1) minimum school leaving age, 2) secondary school past minimum age, and 3) college or university. In NFBC1966 only mother's education was included. Information on severe respiratory infection in early childhood was defined as before age five years in ECRHS *(“Did you have a serious respiratory infection before the age of five years?*”), and within the first year of life in NFBC1966 (“*Did you have serious respiratory infection within the first year of life?*”) (Table 1). These two measures were pooled in the combined analysis. Age at menarche among female offspring was categorized as early (<12 years), normal (12–14 years), and late (>14 years).^25^

### Adult risk factors: smoking and asthma symptom score

The participant's own smoking status was categorized as never/ever smoker or current smoker, and by pack-years (never-smoker, up to 10 pack-years, 10–20 pack-years and >20 pack-years). Having respiratory symptoms during the previous 12 months (ECRHS) were summarized by a 0–5 range asthma symptom score based on the number of symptoms, including: (i) breathless while wheezing, (ii) woken up with a feeling of chest tightness, (iii) attack of shortness of breath at rest, (iv) attack of shortness of breath after exercise, and (v) woken by attack of shortness of breath.^26^ Reporting 3 or more of these respiratory symptoms was used as a threshold in the analyses (a proxy of active asthma). Since we from NFCB1966 only had information on whether the participants had asthma (no/yes), and no information on symptoms, the analysis was restricted to ECRHS.

### Statistical analysis

Associations between early life risk factors and decline in lung function were estimated using mixed effects linear models with random intercepts for subjects nested within centers, including all available data at each time point. The observations included were annual change (Δ) (change/number of years between examinations) in FEV_1_ and FVC between 1st (baseline) and 2nd examination (first follow-up), and between 2nd and 3rd examination (second follow-up, for ECRHS) (Figure S1). Where the 2nd examination was lacking in ECRHS, the change between 1st and 3rd examination was used. For each participant, the mean age at baseline and follow-up was used to model the decline in lung function according to age. A positive value of the coefficients for the covariates in this model represents a decline. That is; a positive coefficient for a particular early life risk factor indicates that the natural age decline in lung function gets steeper with increasing age for individuals exposed to this risk factor. The coefficient represents the estimate of how much steeper the decline is per year, termed mean annual accelerated lung function decline. Missing cases were removed before performing the descriptive analysis.

We ran separate regression analyses including each of the early life risk factors as fixed factors and the participant's id number and study center included as random effects. Since all data were analyzed on individual level, the relative weight for each center is reflected in the number of participants in each center. Finland was considered as one center. All analyses were adjusted for fixed effects of sex, age, height, and lung function at baseline (model 1). All analyses were repeated with further adjustment for personal smoking (pack-years until end of each follow-up; model 2). Finally, we ran a regression analysis including simultaneously the early life risk factors being statistically significant in model 1 and 2 (model 3).

To test whether associations of early life risk factors being statistically significant in the models for ΔFEV_1_ and/or ΔFVC could be modified by the effect of the participant's own current smoking, asthma symptom score, sex, and cohort, we repeated the analysis in model 1 including the relevant interaction terms.

While some of the early life risk factors (mother's smoking during pregnancy and parental asthma) was reported directly by mothers during pregnancy in NFBC1966, it was reported retrospectively by participants in ECRHS. In addition to the interaction analysis by cohort, we therefore included a stratified analysis by cohort for the association between all the early life risk factors we had information on from both cohorts and lung function decline. Since adult obesity has been reported to be associated both with a low FVC and early-menarche,^27^ we tested whether BMI at baseline affected the association between early menarche and FVC decline. We also tested whether an asthma symptom score ≥3 affected the association between parental asthma and participants' decline in FEV_1_ and FVC. Further, we also tested whether the predictors being statistically significant for an accelerated decline in FEV_1_ or FVC also affected the decline in FEV_1_/FVC ratio. To assess whether participants that did not yet had attained their maximal lung function affected the effect estimates, a sensitivity analysis was performed by including only participants above 25 years of age.

Data were analyzed using the software package STATA version 14.0 (StataCorp LP, Texas, USA).

### Role of the funding source

The funding sources did not have any role in designing the study, the collection, analysis, and interpretation of data, in the writing of the manuscript or in the decision to submit the paper for publication.

## Results

### Study population

Table 1 shows prevalence of early life risk factors and adult exposure at baseline. Figure S2 shows unadjusted lung function values in liters (a and b) and mean annual change in ml (c and d) for FEV_1_ and FVC. Overall annual decline in FEV_1_ and FVC was 34.8 ml (SD = 27.3) and 24.2 ml (SD = 33.6), respectively. The decline became steeper with increasing age (Figure S2). Annual decline in FEV_1_ showed an accelerated loss of 1.35 ml (95% CI 1.24, 1.46) per year for men and 1.30 ml (95% CI 1.22, 1.38) per year for women. The corresponding annual accelerated loss in FVC was 1.41 ml (95% CI 1.41, 1.55) for men and 1.43 ml (95% CI 1.34, 1.53) for women.

### Early life risk factors and decline in FEV_1_

By including each early life risk factor separately into the model, statistically significant accelerated annual decline for FEV_1_ of 2.3 ml (95% CI: 0.7, 3.8) was found for participants with a mother that smoked during pregnancy, and 2.6 ml (95% CI: 0.9, 4.4) per year for participants having a mother with asthma, compared to participants without these early life risk factors (model 1, Table 2). For participants that had suffered severe respiratory infection prior to five years of age or that reported early menarche (<12 years of age), an accelerated decline of 1.6 ml per year was of borderline significance for both factors. No statistically significant associations were found for father's smoking during childhood, father's asthma or for any of the other investigated variables. When excluding young participants at baseline (n = 1034, 8.3%), estimates increased marginally for having a mother that smoked during pregnancy (ΔFEV_1_ = 2.5 ml; 95% CI: 0.9, 4.2) and an asthmatic mother (ΔFEV_1_ = 3.0 ml; 95% CI: 1.2, 4.8). Further, to assess whether the number of follow-ups might have affected the precision of the effect of mother's smoking during pregnancy or mother's asthma, we repeated the analysis including only participants having 3 spirometric measurements (3291 participants). The estimate for mother's smoking during pregnancy was somewhat weakened (from 2.3 to 1.7; 95% CI: −0.09, 4.3), while the estimate for mother's asthma increased (from 2.6 to 3.5; 95% CI 0.59, 6.4).

**Table 2:** Change in FEV_1_ from ECHRS1 to ECRHS2, ECRHS2 to ECRHS3, and NFBC1966 I to NFBC1966 II.

Early life risk factors	Δ FEV_1_ (in ml per unit per year) Model 1 (sex, age, height and FEV_1_ at baseline)			Δ FEV_1_ (in ml per unit per year) Model 2 (sex, age, height, FEV_1_, and personal smoking)		
	β	95% CI	p-value	β	95% CI	p-value
Mother's age at birth:						
Age ≤19 years	Ref.			Ref.		
Age 20 through 24 years	−1.20	−3.5, 1.15	0.34	−0.11	−2.48, 2.26	0.67
Age 25 through 29 years	−1.96	−4.3, 0.36		−0.83	−3.17, 1.52	p-value for trend = 0.27
Age 30 through 34 years	−2.27	−4.6, 0.10	p-value for trend = 0.15	−1.18	−3.56, 1.22	
Age 35 through 39 years	−1.39	−3.9, 1.1		−0.44	−3.00, 2.11	
Age ≥ 40 years	−2.25	−5.1, 0.62		−1.08	−4.00, 1.82	
Mother smoked during pregnancy						
No	Ref.			Ref.		
Yes	2.26	0.68, 3.8	0.005	1.64	0.06, 3.22	0.042
Father smoked during childhood (ECRHS)						
No	Ref.			Ref.		
Yes	0.63	−0.57, 1.8	0.30	0.11	−1.11, 1.35	0.86
Caesarean section (ECRHS)						
No	Ref.			Ref.		
Yes	3.16	−0.50, 6.81	0.090	3.49	−0.29, 7.27	0.071
Season of birth						
Other seasons	Ref.			Ref.		
Winter	−0.08	−1.1, 0.96	0.88	0.21	−0.83, 1.3	0.69
Mother having asthma						
No	Ref.			Ref.		
Yes	2.60	0.85, 4.36	0.004	2.26	0.51, 4.02	0.011
Father having asthma						
No	Ref.			Ref.		
Yes	1.48	−0.33, 3.3	0.11	0.99	−0.84, 2.8	0.29
Severe respiratory infection <5 years^a^						
No	Ref.			Ref.		
Yes	1.62	−0.06, 3.3	0.059	1.82	0.11, 3.53	0.037
Age at menarche						
Early (<12 years)	1.57	−0.03, 3.2	0.055	1.40	−0.2, 3.0	0.085
Normal (12–14 years)	Ref.			Ref.		
Late (>14 years)	0.00	−1.7, 1.7	0.99	0.30	−1.5, 2.0	0.74
Mother's education level						
Minimum school leaving age	Ref.			Ref.		
Secondary school	−0.58	−1.7, 0.53	0.31	−0.41	−1.5, 0.69	0.47
College or university	0.0	−1.7, 1.7	0.997	0.10	−1.6, 1.8	0.91
Father's education level (ECRHS)						
Minimum school leaving age	Ref.			Ref.		
Secondary school	1.33	−0.15, 2.8	0.079	1.41	−0.09, 2.9	0.066
College or university	0.80	−0.83, 2.43	0.34	0.94	−0.72, 2.6	0.27

The estimates are adjusted for sex, age, height and FEV_1_ at baseline in model 1. In model 2 the estimates are also adjusted for personal smoking (pack years, continuous). A positive number implies an accelerated decline in FEV_1_ (ml per year) compared to referents, while a negative number implies a lower decline.

a Includes both cohorts: NFBC (within 1 year of age) + ECRHS (within 5 years of age).

Personal smoking was associated with mean annual accelerated decline in FEV_1_ of 2.1 ml (95% CI: 1.8, 2.4) per 10 pack-years of smoking. Adjustment for pack-years at follow-up slightly attenuated the effect estimates for mother's smoking during pregnancy, mother's asthma, and early menarche, while the effect estimate for respiratory infections were slightly strengthened (model 2, Table 2). Using the number of pack-years smoked during follow-up rather than during the whole lifespan did not change the results. Stratifying on never and ever smokers, we found no significant difference in the effect estimate of mother's smoking during pregnancy between never smokers and ever smokers with an estimate of 2.3 (95% CI: 0.0, 4.7) for never smokers (n = 4611) and 2.0 (95% CI: 0.0, 4.1) for ever smokers (n = 7322) (p-value_interaction_ = 0.54). There was a statistically significant interaction between personal smoking (never vs. ever) and reporting respiratory infection within the age of five years (p-value_interaction_ = 0.028), with the negative effect of respiratory infection in early childhood found only among ever-smokers (2.9 vs. 0.1).

Adult asthma symptom score ≥3 was associated with an annual accelerated decline in FEV_1_ of 2.9 ml (95% CI: 0.6, 5.2). We found an interaction between asthma symptom score ≥3 and reporting early age at menarche (p-values_interaction_ = 0.037) with the negative effect of early age at menarche present only among individuals (n = 225) with asthma symptom score ≥3 (8.5 vs. 0.1). No significant interactions were found between the adult exposures (smoking-never vs. smoking ever; asthma symptom score ≥3) and the early life risk factors for FEV_1_-decline i.e. having a mother that smoked during pregnancy and having a mother with asthma (p-values_interaction_ ranging between 0.12 and 0.54). Adjusting the association between having an asthmatic mother and FEV_1_ decline for participants’ asthma symptom score at baseline did not materially change the estimate (from 2.6 to 2.5; 95% CI: 0.8, 3.2).

A final model that mutually adjusted for the early life risk factors that were statistically significant in model 1 or 2 (mother smoking during pregnancy, having a mother with asthma, and severe respiratory infection during childhood) showed only minor changes in the estimates (Table 3).

**Table 3:** Change in FEV_1_ and FVC mutually adjusted for early life risk factors.

Early life risk factors	Δ FEV_1_ (in ml per unit per year) Model 2 (sex, age, height, FEV_1_, and personal smoking)			Δ FEV_1_ (in ml per unit per year) Model 3 (sex, age, height, FEV_1_, and personal smoking, mother smoking, mother asthma and respiratory infection)			Δ FVC (in ml per unit per year) Model 2 (sex, age, height, FVC, and personal smoking)			Δ FVC (in ml per unit per year) Model 3 (sex, age, height, FVC, and personal smoking, mother smoking and father asthma)			Δ FVC (in ml per unit per year) Model 3 (sex, age, height, FVC, and personal smoking, mother smoking, father asthma and early age at menarche)—females only		
	β	95% CI	p-value	β	95% CI	p-value	β	95% CI	p-value	β	95% CI	p-value	β	95% CI	p-value
Mother smoked during pregnancy															
No	Ref						Ref								
Yes	1.64	0.06, 3.22	0.042	1.51	−0.07, 3.1	0.062	1.68	−0.31, 3.7	0.098	1.57	−0.42, 3.56	0.12	1.30	−0.98, 3.58	0.26
Mother having asthma															
No	Ref						–	–	–	–	–	–	–	–	–
Yes	2.23	0.48, 3.98	0.013	2.11	0.35, 3.86	0.018									
Severe respiratory infection <5 years^a^															
No	Ref						–	–	–	–	–	–	–	–	–
Yes	1.82	0.11, 3.53	0.037	1.69	−0.03, 3.40	0.054									
Father having asthma															
No	–	–	–	–	–	–	Ref								
Yes							2.30	0.0, 4.60	0.050	2.30	0.00, 4.60	0.050	0.78	−1.9, 3.5	0.57
Age at menarche															
Normal (12–14 years)	–	–	–	–	–	–	Ref			–	–	–	Ref		
Early (<12 years)							2.36	0.36, 4.37	0.021				2.31	0.30, 4.32	0.024

Change in FEV_1_ and FVC using a linear regression model mutually adjusting for the early life risk factors found to be statistically significant in model 1 or 2 (adjusted for personal smoking). The analysis in model 2 and model 3 are adjusted for sex, age, height, lung function (FEV_1_ or FVC) at baseline, and personal smoking (pack years, continuous). A positive number implies an accelerated decline in FEV_1_ (ml per year) compared to referents, while a negative number implies a lower decline.

a Includes both cohorts: NFBC (within 1 year of age) + ECRHS (within 5 years of age).

### Early life risk factors and decline in FVC

There was an accelerated annual decline in FVC of 2.2 ml (95% CI: 0.2, 4.2) for participants with a mother who smoked during pregnancy, and 2.7 ml (95% CI: 0.5, 5.0) for participants having a father with asthma (model 1, Table 4). Early menarche (<12 years of age) was associated with an annual accelerated decline in FVC of 2.4 ml (95% CI: 0.4, 4.4). In a separate analysis we found a marked association between adult BMI and decline in FVC. When adjusting the association between early menarche and decline in FVC for BMI at baseline (adulthood) the estimate was reduced (from 2.4 to 1.4) and no longer statistically significant (95% CI: −0.7, 3.4). Further, when repeating the analysis including only participants having 3 spirometric measurements, the estimates were somewhat weakened both for having a mother that smoked during pregnancy (from 2.2 to 1.8; 95% CI: −1.4, 5.0) and for reporting early menarche (2.4 to 1.5; 95% CI: −1.4, 4.4). The association between having a father with asthma and decline in FVC was strengthened (from 2.7 to 4.0 (95% CI: 0.5, 7.5). No statistically significant effect on FVC-decline was found for having a mother with asthma or for the other investigated early life risk factors.

**Table 4:** Change in FVC from ECHRS1 to ECRHS2, ECRHS2 to ECRHS3, and NFBC1966 I to NFBC1966 II.

Early life risk factors	Δ FVC (in ml per unit per year) Model 1 (sex, age, height and FVC at baseline)			Δ FVC (in ml per unit per year) Model 2 (sex, age, height, FVC, and personal smoking)		
	β	95% CI	p-value	β	95% CI	p-value
Mother's age at birth:						
Age ≤19 years	Ref.		0.20	Ref.		0.46
Age 20 through 24 years	−1.33	−4.28, 1.61		−0.75	−3.7, 2.2	
Age 25 through 29 years	−2.67	−5.59, 0.24		−1.82	−4.8, 1.1	p-value for trend = 0.40
Age 30 through 34 years	−2.65	−5.63, 0.33	p-value for trend = 0.22	−1.86	−4.9, 1.2	
Age 35 through 39 years	−1.24	−4.41, 1.93		−0.51	−3.7, 2.7	
Age ≥ 40 years	−2.98	−6.59, 0.63		−2.14	−5.8, 1.5	
Mother smoked during pregnancy						
No	Ref.			Ref.		
Yes	2.18	0.20, 4.20	0.031	1.7	−0.31, 3.7	0.098
Father smoked during childhood (ECRHS)						
No	Ref.			Ref.		
Yes	1.16	−0.36, 2.7	0.14	0.87	−0.59, 2.4	0.27
Caesarean section (ECRHS)						
No	Ref.			Ref.		
Yes	2.79	−1.8, 7.4	0.24	2.91	−1.9, 7.7	0.24
Season of birth						
Other seasons	Ref.			Ref.		
Winter	0.36	−0.95, 1.7	0.59	0.59	−0.73, 1.9	0.88
Mother having asthma						
No	Ref.			Ref.		
Yes	1.75	−0.45, 4.0	0.12	1.46	−0.8, 3.7	0.20
Father having asthma						
No	Ref.			Ref.		
Yes	2.74	0.47, 5.0	0.018	2.30	0.0, 4.6	0.050
Severe respiratory infection <5 years^a^						
No	Ref.			Ref.		
Yes	1.53	−0.59, 3.65	0.16	1.39	−0.77, 3.6	0.21
Age at menarche						
Early (<12 years)	2.43	0.4, 4.4	0.018	2.36	0.4, 4.4	0.021
Normal (12–14 years)	Ref.			Ref		
Late (>14 years)	−0.08	−3.0, 1.4	0.46	−0.43	−2.7, 1.8	0.70
Mother's education level						
Minimum school leaving age	Ref.			Ref.		
Secondary school	−1.4	−2.8, 0.0	0.051	−1.29	−2.7, 0.11	0.07
College or university	−1.1	−3.3, 1.1	0.34	−1.05	−3.2, 1.2	0.35
Father's education level (ECRHS)						
Minimum school leaving age	Ref.			Ref.		
Secondary school	−0.32	−2.2, 1.56	0.74	−0.44	−2.3, 1.5	0.65
College or university	−1.3	−3.4, 0.76	0.22	−1.17	−3.3, 0.9	0.28

The estimates are adjusted on sex, age, height and FVC at baseline in model 1. In model 2 the estimates are also adjusted for adult smoking (pack years, continuous). A positive number implies an accelerated decline in FVC compared to referents, while a negative number implies a lower decline.

a Includes both cohorts: NFBC (within 1 year of age) + ECRHS (within 5 years of age).

Personal smoking per 10 pack-years was associated with mean annual accelerated decline in FVC of 1.7 ml (95% CI: 1.3, 2.1). The effect of having a father with asthma and early menarche were practically unaltered after adjustment for personal smoking, while the association with maternal smoking was attenuated and no longer statistically significant. Using the number of pack-years smoked during follow-up rather than during the whole lifespan did not change the results. Stratifying on never and ever smokers, the effect estimate of having a mother that smoked during pregnancy was 3.1 (95% CI: 0.0, 6.2) for never smokers (n = 4435) and 1.4 (95% CI: −1.1, 4.0) for ever smokers (n = 7036) (p-value_interaction_ p = 0.28). We found a significant interaction between personal smoking (never vs. ever) and respiratory infections in early childhood (p-values_interaction_ = 0.026), with the negative effect of respiratory infections in early childhood found only among ever-smokers (3.0 vs. −0.1). No other interaction effects were found (p-values_interaction_ ranging between 0.28 and 0.67). An asthma symptom score of ≥3 was associated with accelerated decline in FVC (3.6 ml per year; 95% CI: 0.7, 6.4).

A final model mutually adjusted for early life risk factors being statistically significant in model 1 or 2 (mother smoking during pregnancy, having a father with asthma, and early age at menarche) did not change the estimates for the association between accelerated decline in FVC for the early risk factors having a father with asthma and early age at menarche (Table 3). Adjusting for participants asthma at baseline (asthma symptom score ≥3) did not materially change the estimate of the association between having a father with asthma and accelerated FVC decline (2.7; 95% CI: 0.4, 4.9).

### Early life risk factors and decline in FEV_1_/FVC

Mean annual reduction in FEV_1_/FVC was 0.33% (SD 0.45) and 0.38% (SD 0.45) in males and females, respectively (p-value for difference between sex <0.001). Having a mother with asthma was associated with an accelerated decline in FEV_1_/FVC of 0.036 per year (95% CI: 0.04, 0.7). No statistically significant effect on the age-related decline in FEV_1_/FVC-ratio was found for any of the other early life factors affecting lung function (mother smoking during pregnancy, having a father with asthma, severe respiratory infection, early menarche, or Caesarean section).

### Sex-specific patterns

The associations between ΔFEV_1_ and having a mother that smoked or had asthma appeared to be slightly stronger and statistically significant only in women (Tables S2 and S3, model 1 and 2). Accordingly, father's asthma was associated with accelerated decline in both FEV_1_ and FVC in men, but not women (Tables S2–S5). Men, but not women, delivered by Caesarean section had an annual accelerated decline in FEV_1_ (FEV_1_ = 6.7 ml; 95% CI: 0.7, 12.7) (Tables S2 and S3). Nevertheless, there was no statistically significant differences between men and women for the associations of maternal smoking, maternal asthma, paternal asthma, or Caesarean section with FEV_1_ decline (p-values_interaction_ 0.18–0.93) and FVC decline (p-values_interaction_ 0.44–0.80).

### Cohort-specific patterns

There was no significant interaction between the two cohorts and the variables with potential differential recall bias in the two cohorts, such as mother smoking during pregnancy and parental asthma (reported by participant or parents), for neither ΔFEV_1_ (p-values_interaction_ ranging from 0.29 to 0.14) nor ΔFVC (p-values_interaction_ ranging from 0.97 to 0.37). Since the information on these early risk factors was retrospectively ascertained in the ECRHS and prospectively ascertained in NFBC1966, we nevertheless estimated the association between the early life factors investigated and decline in FEV_1_ and FVC, respectively, stratified by cohort (Tables S6 and S7). The associations were in the same direction for both cohorts, but generally stronger in the NFBC1966 for mother smoking during pregnancy compared to the ECRHS. For parental asthma the associations with lung function decline were stronger in ECRHS compared to NFBC1966.

## Discussion

By pooling two large prospective cohorts comprising more than 11,000 adult participants with a mean follow-up time of more than 20 years, we found evidence for similar associations between early life risk factors and accelerated decline into the seventh decade for both FEV_1_ and FVC. Accelerated decline in FEV_1_ was markedly associated with mother's smoking during pregnancy, mother's asthma, and severe respiratory infection in early childhood. Accelerated decline in FVC was associated with mother's smoking during pregnancy, father's asthma, and early menarche. Each of the effect estimates of mother's smoking, parental asthma and early menarche were comparable to the impact of personal smoking of 10 pack-years. Only mother's asthma was associated with a decline in FEV_1_/FVC. Overall, effect estimates were largely consistent after further adjustment for personal smoking and in mutual adjustment for other early life risk factors. No associations between lung function decline and mothers age at delivery, being born during winter, being delivered by Caesarean section, or parental education were identified.

Having a mother that smoked during pregnancy, but not a father that smoked in early childhood, was a strong predictor of accelerated decline in FEV_1_ and FVC. Effect sizes were comparable to those of personal smoking of 10 pack-years (2.1 ml and 1.7 ml per year, respectively). This corresponds to an increased rate in FEV_1_ and FVC decline of 4.3% and 6.5%, respectively, of the overall annual decline in lung function. Mother's smoking was associated with FEV_1_-decline in previous analyses of sub-samples of the present study population, with an effect of mother's smoking during pregnancy or childhood of 1.3 ml per year (95% CI: −0.6, 3.2)^9^ and 1.8 ml per year (95% CI: 0.3, 3.3).^11^ Further, mother's heavy smoking at age seven (no similar data on smoking in pregnancy) was reported to be associated with a lung function trajectory with accelerated decline in FEV_1_.^13^ With consistent estimates both with and without mutual adjustments of other early risk factors associated with an accelerated lung function decline, a large study population and long follow-up time, and analysis of FVC as well as FEV_1_, our current study gives important evidence that the adverse effects induced by mother's smoking during pregnancy continues to influence the ageing lung decades later.

Participants who suffered severe respiratory infection prior to five years of age had an accelerated decline in FEV_1_. For both FEV_1_ and FVC there was a statistically significant interaction with smoking showing a negative impact of childhood infection only among ever-smokers. Although lower respiratory tract infections in early childhood have been reported to be associated both with a lower lung function in adulthood^9^^,^^13^^,^^28^, ^29^, ^30^, ^31^ and a lung function trajectory with accelerated decline in FEV_1_,^13^ several studies have reported on a lack of association.^9^^,^^11^^,^^28^^,^^29^ It is likely that respiratory infections occurring within the first years of life, at the same time as the development of the immune and respiratory system is at its’ most active, might impose a consecutive susceptibility of the lung that persists through adolescence and adulthood.^32^ This susceptibility might accentuate the observed smoking-related accelerated decline.

Having a mother with asthma was found to contribute to accelerated decline in both FEV_1_ and FEV_1_/FVC ratio, while father's asthma was associated with a decline in FVC. The estimate of mother's asthma on FEV_1_ decline and father's asthma on FVC decline were both somewhat attenuated, but still statistically significant, when adjusted for participants' asthma symptoms at baseline (asthma symptom score ≥3). Having a mother with asthma has previously been reported to be associated with a persistently low lung function trajectory (FEV_1_ and FEV_1_/FVC ratio) in the Tucson Children's Respiratory Study,^30^ while parental asthma (combined) was non-significantly associated with the lung function trajectory “early below average, accelerated decline” measured as FEV_1_ in the TAHS study.^13^ In the TAHS study it was also found that subjects on both a low FEV_1_/FVC ratio trajectory and low FVC trajectory had the highest prevalence of COPD and other respiratory symptoms at age 53 years, but also parental asthma and childhood respiratory illnesses.^16^ Overall, the observations in the present study support the association of having a mother with asthma and an accelerated decline in lung function measured as FEV_1_ and FEV_1_/FVC ratio. Although the associations of early life factors with lung function decline overall did not differ according to sex, the finding of a stronger father–son associations for having a father with asthma and accelerated FVC decline, deserve further investigation. This sex-specific patterns together with a stronger mother–daughter associations for maternal smoking are compatible with epigenetically mediated underlying mechanisms.^33^

Early menarche was associated with accelerated decline in FVC. When adjusting for BMI at baseline the effect of early menarche was reduced (from 2.4 to 1.3) and no longer significant. This can be interpreted as the effect of early menarche on FVC at least partly being mediated through, or confounded by, obesity. Early age at menarche has previously been linked to general poorer lung health in terms of lower FVC and FEV_1_,^10^^,^^14^^,^^22^ FEV_1_/FVC,^34^ lower functional residual capacity,^14^ and excess risk of developing asthma.^10^^,^^25^^,^^35^ In a study examining the association between early age of menarche (<12 years) and decline of FEV_1_ and FVC (age 45–53 years) a non-statistically annual decline of 1.6 (−9.1, 6.0) and 3.5 ml (−6.3, 13.4) was reported.^14^ Of note the follow-up time was substantially shorter and study population smaller than in the current analysis. A larger effect of early menarche on FVC than on FEV_1_ is supported by a Mendelian randomization analysis of poly-cystic ovary syndrome, which often occurs in women with early menarche, suggesting a causal relationship with FVC, but not with FEV_1_.^36^ Overall, our results of accelerated decline in FVC supports a link between early age at menarche and lung ageing, although the causality and possible mechanisms remains to be elucidated.

Early life risk factors have been suggested to increase susceptibility to adult smoking.^11^^,^^35^, ^36^, ^37^ Dratva et al.^11^ reported stronger effects of early life risk factors on FEV_1_ decline among smokers. Allison et al.^37^ found lower FEV_1_ and FVC at 43 years in persons with early life risk factors if they smoked, but no effect on decline from 42 to 60–64 years of age. One study reported that mother's smoking accentuated the effect of adult personal smoking on FEV_1_-decline,^38^ while another reported that parental and personal smoking act synergistically on lung function decline in early adulthood.^39^ In the present study personal smoking on its own was associated with a marked accelerated decline in both FEV_1_ and FVC, but we did not find statistically significant interactions between personal and mother's smoking in the associations with lung function decline. The estimates were largely consistent after adjustment for personal smoking, and when stratifying analysis on never and ever smokers. An adult asthma symptom score ≥3 was associated with accelerated decline in FEV_1_ and FVC, compatible with previous studies showing a lung function trajectory with accelerated decline in asthmatics.^40^, ^41^, ^42^, ^43^ Nevertheless, we did not find any significant interaction between an asthma symptom score ≥3 and early life risk factors associated with lung function decline.

Main strengths of the present study include the prospective design with measurements of both FEV_1_ and FVC at up to three time points after the plateau phase, the large sample size, long follow-up time, and extensive information on early life risk factors, potential confounders, and other covariates. One limitation is risk of loss to follow-up and recall-bias. However, in an overlapping cohort of ECRHS (Respiratory Health in Northern Europe, RHINE) estimates of most exposure-outcome associations for prevalence of self-reported respiratory outcomes were unbiased,^44^ and a good agreement between offspring and parents’ reporting of parental asthma was found.^45^ The two cohorts differed in the method of collection of information on early life risk factors, but no significant interaction between cohort and variables with a potential differential recall bias for neither outcome was found. Since we only had post-bronchodilator spirometry values measured for ECRHS and then only at one point in time (ECRHS3), the pre-bronchodilator spirometry values were used in the analysis. However, although post-bronchodilator lung function measures are recommended for the diagnosis of COPD and suggested to be preferred in predicting mortality,^46^ the aim in the present study was to describe the association between early life factors and later lung function decline rather than describing the clinical pathology. Another limitation was lack of information on exposure to ambient and indoor air pollutants.

In conclusion, after taking personal smoking into account, having a mother that smoked during pregnancy, parental asthma, and early menarche were associated with accelerated lung function decline in adulthood comparable to the impact of personal smoking of 10 pack-years. This study of early life risk factors as related to decline in both FEV_1_ and FVC in a large study population followed over more than twenty years provides solid evidence of an influence of these factors on changes in lung function over a lifespan.

## Contributors

JK, TR and CS developed the concept for the study. CS, DJ and SCD contributed to the project design and funding acquisition of the study. MW contributed to the data management, while JK and TR accessed, verified and analyzed the data. All authors (JK, TR, CS, DJ, SCD, MW, SA, A-EC, JD, KAF, JG-A, BL, CJL, FGR, VS, AGC, JH, MH, CJ, BB, RJ, M-RJ) contributed to analytic strategy and interpretation of results. JK and TR drafted the manuscript, and all authors reviewed and revised the manuscript critically for important intellectual content. All authors gave approval of the final version to be published and agreed to be accountable for all aspects of the work.

## Data sharing statement

The ECRHS and NFBC1966 datasets are not publicly available. The data that support the findings of this study are available from the corresponding author upon reasonable request.

## Declaration of interests

SCD and CJL have received an investigator-initiated grant from GSK and a partnership grant from AZ for unrelated work. All other authors declare no conflict of interest.
