# Supplementary material for: Early life exposures contributing to accelerated lung function decline in adulthood – a follow-up study of 11,000 adults from the general population
Source: eClinicalMedicine. 2023 Dec 8;66:102339. doi: 10.1016/j.eclinm.2023.102339 (PMC10714210; doi:10.1016/j.eclinm.2023.102339)
Supplement: Supplementary Figure S1 [file mmc1.docx]

**NFBC1966**

Follow-up study of Finnish children with expected delivery in 1966 (12,058 live births)

**ECRHS stage I**

Postal screening questionnaire (200 682 adults)

ECRHS1 stage II

(1991-93)

Age 20-44 years

N = 5,159

NFBC1966 I

(1997)

31 years

N = 5,812

Lost to follow-up:

N = 1,389

Recruited into the first follow-up: n=321

Lost to follow-up:

N = 1,981

ECRHS2

(1998-2002)

Age 29-55 years

N = 4,091

NFBC1966 II

(2012-13)

Age 45-47 years

N = 3,831

Recruited back to the second follow-up:

N = 1,029

Lost to follow-up from second to third study:

N = 476

ECRHS3

(2010-13)

Age 46-68 years

N = 4,644

***Figure S1:* Flow diagram of study population in ECRHS and NFBC1966.** Study population from ECRHS used in the present study comprise participants from 26 of the originally 54 centers from ECRHS1 Stage I, where N = number of participants with lung function measurements (FEV_1_) in the three studies. More information about the cohorts can be obtained on [www.ecrhs.org](http://www.ecrhs.org) and [www.oulu.fi/nfbc/](http://www.oulu.fi/nfbc/).

adjusted for any other variables.
