# Supplementary material for: Early life exposures contributing to accelerated lung function decline in adulthood – a follow-up study of 11,000 adults from the general population
Source: eClinicalMedicine. 2023 Dec 8;66:102339. doi: 10.1016/j.eclinm.2023.102339 (PMC10714210; doi:10.1016/j.eclinm.2023.102339)
Supplement: Supplementary Figure S2 [file mmc2.docx]

***
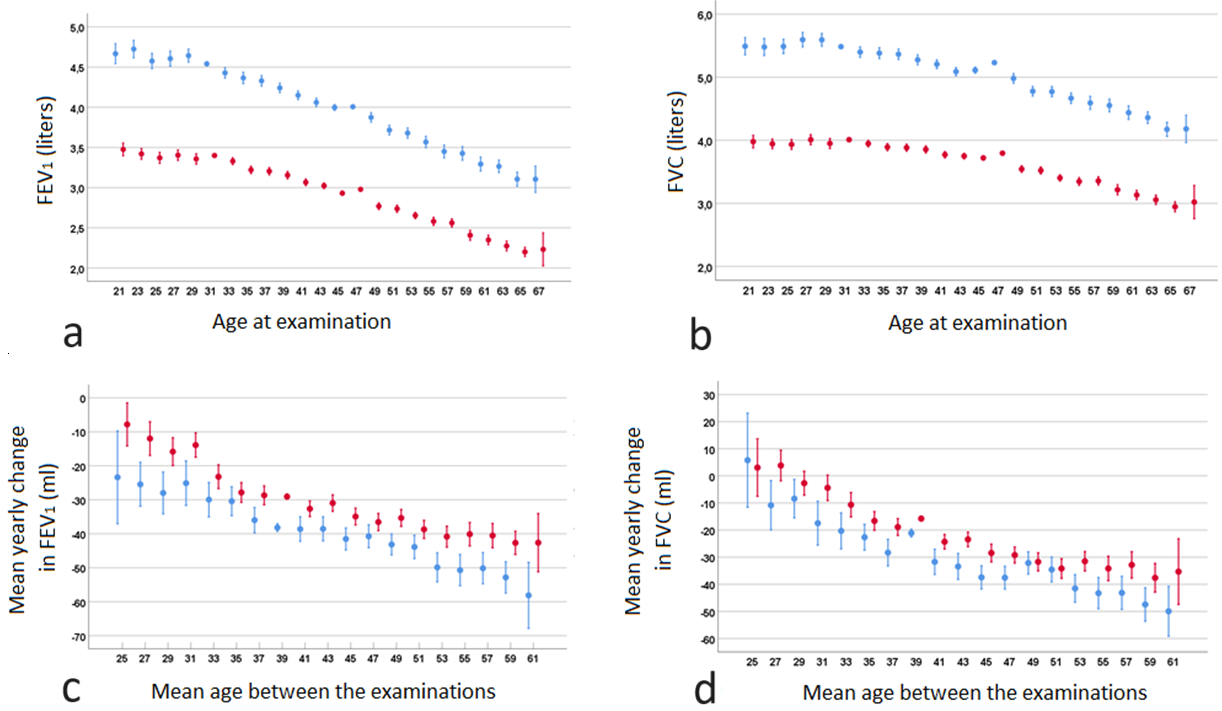
***

***Figure S2* Lung function of participants in ECRHS and NFBC1966 combined.** Crude values (in liters) in FEV_1_ (A) and FVC (B) for men (blue) and women (red) measured at the respective ages of the total cohort and in subsequent two time points, and mean yearly change (in milliliters) in FEV_1_ (C) and FVC (D) for men (blue) and women (red) measured at the respective ages of the total cohort and in subsequent two time points. The data are not adjusted for any other variables.
