# Supplementary material for: Early life exposures contributing to accelerated lung function decline in adulthood – a follow-up study of 11,000 adults from the general population
Source: eClinicalMedicine. 2023 Dec 8;66:102339. doi: 10.1016/j.eclinm.2023.102339 (PMC10714210; doi:10.1016/j.eclinm.2023.102339)
Supplement: Supplementary Table S1 [file mmc3.docx]

| **Country** | **Center** | **FEV_1_** | | | **FVC** | | | **Brand of spirometers** | | |
| --- | --- | --- | --- | --- | --- | --- | --- | --- | --- | --- |
|  |  | **Baseline** | **First follow-up** | **Second follow-up** | **Baseline** | **First follow-up** | **Second follow-up** | **Baseline** | **First**  **follow-up** | **Second follow-up** |
| Belgium | Antwerp South | 130 | 132 | 136 | 131 | 132 | 132 | SensorMedics* | Jaeger Masterscope | NDD EasyOne |
|  | Antwerp City | 140 | 119 | 179 | 139 | 119 | 175 | SensorMedics* | Jaeger Masterscope | NDD EasyOne |
| Denmark | Aarhus | 176 | − | 187 | 178 | − | 183 | Vitalograph | NA | NDD EasyOne |
| Estonia | Tartu | 80 | 63 | 114 | 61 | 45 | 112 | Jaeger Masterscope | Jaeger Masterscope | NDD EasyOne |
| Germany | Hamburg | 268 | 180 | 265 | 252 | 178 | 261 | Jaeger Masterscope | Jaeger Masterscope | NDD EasyOne |
|  | Erfurt | 294 | 193 | 321 | 286 | 193 | 315 | Jaeger Masterscope | Jaeger Masterscope | NDD EasyOne |
| Spain | Barcelona | 107 | 113 | 105 | 107 | 114 | 104 | Biomedin | Biomedin | NDD EasyOne |
|  | Galdakao | 269 | 267 | 263 | 268 | 266 | 261 | Biomedin | Biomedin | NDD EasyOne |
|  | Albacete | 162 | 139 | 161 | 161 | 13 | 161 | Biomedin | Biomedin | NDD EasyOne |
|  | Oviedo | 108 | 120 | 125 | 108 | 119 | 123 | Biomedin | Biomedin | NDD EasyOne |
|  | Huelva | 100 | 96 | 96 | 100 | 97 | 96 | Biomedin | Biomedin | NDD EasyOne |
| France | Bordeaux | 172 | 88 | 111 | 162 | 89 | 108 | Vitalograph | Vitalograph | NDD EasyOne |
|  | Grenoble | 337 | 271 | 240 | 332 | 271 | 236 | Biomedin | Biomedin | NDD EasyOne |
|  | Montpellier | 186 | 97 | 121 | 184 | 96 | 121 | Biomedin | Biomedin | NDD EasyOne |
|  | Paris | 315 | 258 | 244 | 311 | 256 | 228 | Biomedin | Biomedin | NDD EasyOne |
| Italy | Pavia | 63 | 76 | 70 | 63 | 76 | 70 | Biomedin | Biomedin | NDD EasyOne |
|  | Turin | 53 | 57 | 36 | 53 | 57 | 38 | Biomedin | Biomedin | Biomedin |
|  | Verona | 88 | 84 | 81 | 88 | 84 | 74 | Biomedin | Biomedin | Biomedin |
| England | Ipswich | 141 | 118 | 131 | 140 | 118 | 131 | Biomedin | Biomedin | NDD EasyOne |
|  | Norwich | 133 | 111 | 132 | 132 | 111 | 130 | Biomedin | Biomedin | NDD EasyOne |
| Iceland | Reykjavik | 374 | 338 | 344 | 373 | 338 | 336 | SensorMedics* | SensorMedics* | NDD EasyOne |
| Norway | Bergen | 351 | 330 | 349 | 350 | 326 | 344 | SensorMedics* | SensorMedics* | NDD EasyOne |
| Sweden | Gothenburg | 250 | 176 | 202 | 242 | 166 | 202 | SensorMedics* | SensorMedics* | NDD EasyOne |
|  | Umeå | 210 | 176 | 190 | 211 | 171 | 188 | SensorMedics* | SensorMedics* | NDD EasyOne |
|  | Uppsala | 256 | 234 | 246 | 243 | 229 | 246 | SensorMedics* | SensorMedics* | NDD EasyOne |
| Switzerland | Basel | 396 | 314 | 400 | 368 | 300 | 399 | SensorMedics* | SensorMedics* | NDD EasyOne |
| Total ECRHS | | 5,159 | 4,150 | 4,849 | 5,043 | 4,086 | 4,774 |  |  |  |
| NFBC1966 | | 5,812 | 3,831 | - | 5,809 | 3,830 | - | Vitalograph P-model (1997) | Spira MasterScreen Pneumo spirometer (2012) | NA |
| **ECRHS and NFBC1966 combined** | | **10,971** | **7,981** | **4,849** | **10,852** | **7,916** | **4,774** |  |  |  |

* Volume-displacement spirometer, NA = not applicable (did not participate in ECRHS2, first follow-up)

***Table S1:*** **Number of lung function measurements and spirometers used.** Number of participants having the lung function measured (FEV_1_ and FVC) from the respective centers in ECRHS and NFBC1966, and the respective brand of spirometers used. Participants contributing with at least two measurements are included in the analysis.
