# Supplementary material for: Early life exposures contributing to accelerated lung function decline in adulthood – a follow-up study of 11,000 adults from the general population
Source: eClinicalMedicine. 2023 Dec 8;66:102339. doi: 10.1016/j.eclinm.2023.102339 (PMC10714210; doi:10.1016/j.eclinm.2023.102339)
Supplement: Supplementary Table S2 [file mmc4.docx]

| **Early life risk factors** | **Δ FEV_1_ (in ml per unit per year)** | | | | | |
| --- | --- | --- | --- | --- | --- | --- |
|  | **Males** | | | **Females** | | |
|  | β | 95% CI | p-value | β | 95% CI | p-value |
| Mother’s age at birth  *Age ≤19 years*  *Age 20 through 24 years*  *Age 25 through 29 years*  *Age 30 through 34 years*  *Age 35 through 39 years*  *Age ≥ 40 years* | Ref.  -0⋅65  -2⋅05  -2⋅53  -1⋅33  -1⋅16 | -4⋅7, 3⋅4  -6⋅1, 2⋅0  -6⋅6, 1⋅5  -5⋅7, 3⋅0  -6⋅9, 3⋅8 | 0⋅57  p-value for trend  = 0⋅38 | Ref.  -1⋅61  -1⋅92  -2⋅08  -1⋅45  -3⋅26 | -4⋅3, 1⋅1  -4⋅6, 0⋅7  -4⋅8, 0⋅6  -4⋅3, 1⋅4  -6⋅5, 0⋅0 | 0⋅49  p-value for trend  = 0⋅19 |
| Mother smoked during pregnancy  *No*  *Yes* | Ref.  1⋅95 | -1⋅0, 4⋅9 | 0⋅19 | Ref.  2⋅23 | 0⋅5, 3⋅9 | 0⋅011 |
| Father smoked during childhood  *No*  *Yes* | Ref.  1⋅87 | -0⋅1, 3⋅9 | 0⋅64 | Ref.  -0⋅67 | -2⋅1, 0⋅7 | 0⋅35 |
| Caesarean section*  *No*  *Yes* | Ref.  6⋅71 | 0⋅7, 12⋅7 | 0⋅028 | -0⋅05 | -4⋅4, 4⋅3 | 0⋅98 |
| Season of birth  *Other seasons*  *Winter* | Ref.  -0⋅70 | -2⋅4, 1⋅0 | 0⋅43 | Ref.  0⋅29 | -0⋅9, 1⋅5 | 0⋅64 |
| Mother having asthma  *No*  *Yes* | Ref.  2⋅38 | -0⋅9, 5⋅6 | 0⋅15 | Ref.  2⋅72 | 0⋅8, 4⋅6 | 0⋅005 |
| Father having asthma  *No*  *Yes* | Ref.  3⋅88 | 0⋅6, 7⋅1 | 0⋅019 | Ref.  0⋅04 | -2⋅0, 2⋅06 | 0⋅97 |
| Severe respiratory infection < 5 years  *No*  *Yes* | Ref.  3⋅05 | -0⋅3, 6⋅4 | 0⋅071 | Ref.  0⋅62 | -1⋅6, 2⋅8 | 0⋅58 |
| Mother’s education level  *Minimum school leaving age*  *Secondary school*  *College or university* | Ref.  -1⋅32  0⋅60 | -3⋅2, 0⋅6  -2⋅3, 3⋅5 | 0⋅17  0⋅68 | Ref.  -0⋅07  -0⋅65 | -1⋅3, 1⋅2  -2⋅7, 1⋅4 | 0⋅91  0⋅53 |
| Father’s education level  *Minimum school leaving age*  *Secondary school*  *College or university* | Ref.  1⋅14  1⋅71 | -1⋅3, 3⋅6  -1⋅0, 4⋅4 | 0⋅36  0⋅22 | Ref.  1⋅33  0⋅02 | -0⋅4, 3⋅0  -1⋅9, 1⋅9 | 0⋅13  0⋅98 |

*Based on 144 participants (2.6%) delivered by Caeserian section; 73 males and 71 females.

***Table S2:*** **Change in FEV_1_ stratified by sex (model 1).** Change in FEV_1_ (Δ FEV_1_ = in ml per unit per year) from wave 1 to 2, 2 to 3 and 1 to 3, stratified by sex. The estimates are adjusted for age, height and FEV_1_ at baseline (ECRHS1 / NFBC1966 I) (model 1).
