# Supplementary material for: Early life exposures contributing to accelerated lung function decline in adulthood – a follow-up study of 11,000 adults from the general population
Source: eClinicalMedicine. 2023 Dec 8;66:102339. doi: 10.1016/j.eclinm.2023.102339 (PMC10714210; doi:10.1016/j.eclinm.2023.102339)
Supplement: Supplementary Table S3 [file mmc5.docx]

| **Early life risk factors** | **Δ FEV_1_ (in ml per unit per year)** | | | | | |
| --- | --- | --- | --- | --- | --- | --- |
|  | **Males** | | | **Females** | | |
|  | β | 95% CI | p-value | β | 95% CI | p-value |
| Mother’s age at birth  *Age ≤19 years*  *Age 20 through 24 years*  *Age 25 through 29 years*  *Age 30 through 34 years*  *Age 35 through 39 years*  *Age ≥ 40 years* | Ref.  1⋅43  -0⋅041  -0⋅59  0⋅76  1⋅27 | -2⋅7, 5⋅6  -4⋅2, 4⋅1  -4⋅8, 3⋅6  -3⋅7, 5⋅2  -3⋅7, 6⋅3 | 0⋅58  p-value for trend: 0⋅71 | Ref.  -1⋅39  -1⋅55  -1⋅76  -1⋅48  -3⋅15 | -4⋅1, 1⋅3  -4⋅2, 1⋅1  -4⋅5, 0⋅9  -4⋅4, 1⋅4  -6⋅4, 0⋅1 | 0⋅57  p-value for trend: 0⋅15 |
| Mother smoked during pregnancy  *No*  *Yes* | Ref.  0⋅97 | -2.0, 3⋅9 | 0⋅52 | Ref.  1⋅85 | 0⋅2, 3⋅6 | 0⋅033 |
| Father smoked during childhood  *No*  *Yes* | Ref.  1⋅36 | -0⋅7, 3⋅4 | 0⋅19 | Ref.  -1⋅17 | -2⋅6, 0⋅3 | 0⋅11 |
| Caesarean section*  *No*  *Yes* | Ref.  7⋅92 | 1⋅6, 14⋅2 | 0⋅014 | -0⋅31 | -4⋅7, 4.1 | 0⋅89 |
| Season of birth  *Other seasons*  *Winter* | Ref.  -0⋅57 | -2⋅3, 1⋅2 | 0⋅52 | Ref.  0⋅76 | -0⋅5, 2.0 | 0⋅22 |
| Mother having asthma  *No*  *Yes* | Ref.  1⋅96 | -1⋅3, 5⋅2 | 0⋅24 | Ref.  2⋅44 | 0⋅6, 4⋅3 | 0⋅011 |
| Father having asthma  *No*  *Yes* | Ref.  3⋅54 | 0⋅2, 6⋅9 | 0⋅037 | Ref.  -0⋅52 | -2⋅5, 1⋅5 | 0⋅61 |
| Severe respiratory infection < 5 years  *No*  *Yes* | Ref.  2⋅71 | -0⋅2, 5⋅7 | 0⋅071 | Ref.  0⋅73 | -1⋅2, 2⋅7 | 0⋅47 |
| Mother’s education level  *Minimum school leaving age*  *Secondary school*  *College or university* | Ref.  -0⋅86  1⋅11 | -2⋅7, 1⋅0  -1⋅8, 4⋅0 | 0⋅37  0⋅45 | Ref.  -0⋅11  -0⋅88 | -1⋅4, 1⋅2  -2.9, 1.1 | 0⋅86  0⋅39 |
| Father’s education level  *Minimum school leaving age*  *Secondary school*  *College or university* | Ref.  1⋅36  1⋅97 | -1⋅1, 3⋅9  -0⋅8, 4⋅8 | 0⋅28  0⋅16 | Ref.  1⋅23  0⋅06 | -0⋅5, 3.0  -1⋅9, 2.0 | 0⋅16  0⋅96 |

*Based on 144 participants (2.6%) delivered by Caeserian section; 73 males and 71 females.

***Table S3:*** **Change in FEV_1_ stratified by sex and adjusted for personal smoking (model 2).** Change in FEV_1_ (Δ FEV_1_ = in ml per unit per year) from wave 1 to 2, 2-3 and 1 to 3, stratified on sex. The estimates are adjusted for age, height, FEV_1_ at baseline (ECRHS1 / NFBC1966 I) and personal smoking (pack years) (model 2).
