# Supplementary material for: Early life exposures contributing to accelerated lung function decline in adulthood – a follow-up study of 11,000 adults from the general population
Source: eClinicalMedicine. 2023 Dec 8;66:102339. doi: 10.1016/j.eclinm.2023.102339 (PMC10714210; doi:10.1016/j.eclinm.2023.102339)
Supplement: Supplementary Table S4 [file mmc6.docx]

| **Early life risk factors** | **Δ FVC (in ml per unit per year)** | | | | | |
| --- | --- | --- | --- | --- | --- | --- |
|  | **Males** | | | **Females** | | |
|  | β | 95% CI | p-value | β | 95% CI | p-value |
| Mother’s age at birth  *Age ≤19 years*  *Age 20 through 24 years*  *Age 25 through 29 years*  *Age 30 through 34 years*  *Age 35 through 39 years*  *Age ≥ 40 years* | Ref.  -0⋅55  -1⋅83  -2⋅60  -0⋅11  -2⋅07 | -5⋅7, 4⋅6  -6⋅9, 3⋅3  -7⋅8, 2⋅6  -5.6, 5⋅4  -8⋅3, 4⋅2 | 0⋅64  p-value for trend  = 0⋅54 | Ref.  -2⋅21  -3⋅86  -3⋅07  -2⋅35  -4⋅02 | -5⋅5, 1⋅1  -7⋅1, -0⋅6  -6⋅4, 0⋅3  -5⋅9, 1⋅3  -8⋅1, 0⋅1 | 0⋅17  p-value for trend  = 0⋅21 |
| Mother smoked during pregnancy  *No*  *Yes* | Ref.  2⋅84 | -0⋅8, 6⋅5 | 0⋅13 | Ref.  1⋅59 | -0⋅5, 3⋅7 | 0⋅14 |
| Father smoked during childhood  *No*  *Yes* | Ref.  1⋅76 | -0⋅7, 4⋅3 | 0⋅18 | Ref.  0⋅38 | -1⋅4, 2⋅1 | 0⋅67 |
| Caesarean section*  *No*  *Yes* | Ref.  4⋅83 | -3⋅0, 12⋅6 | 0⋅22 | 1⋅32 | -4⋅0, 6⋅6 | 0⋅63 |
| Season of birth  *Other seasons*  *Winter* | Ref.  -0⋅36 | -2⋅6, 1⋅9 | 0⋅75 | Ref.  1⋅0 | -0⋅5, 2⋅5 | 0⋅19 |
| Mother having asthma  *No*  *Yes* | Ref.  3⋅13 | -1⋅0, 7⋅3 | 0⋅14 | Ref.  0⋅97 | -1⋅4, 3⋅3 | 0⋅43 |
| Father having asthma  *No*  *Yes* | Ref.  4⋅2 | 0⋅1, 8⋅4 | 0⋅043 | Ref.  2⋅14 | -0⋅4, 4⋅6 | 0⋅093 |
| Severe respiratory infection < 5 years  *No*  *Yes* | Ref.  3⋅64 | -0⋅6, 7⋅9 | 0⋅094 | Ref.  0⋅74 | -2⋅0, 3⋅5 | 0⋅59 |
| Mother’s education level  *Minimum school leaving age*  *Secondary school*  *College or university* | Ref.  -1⋅9  -0⋅40 | - 4⋅3, 0⋅43  - 4⋅1, 3⋅3 | 0⋅11  0⋅83 | Ref.  -1⋅15  -1⋅80 | -2⋅7, 0⋅4  -4⋅4, 0⋅74 | 0⋅15  0⋅17 |
| Father’s education level  *Minimum school leaving age*  *Secondary school*  *College or university* | Ref.  -1⋅42  -1⋅40 | -4⋅6, 1⋅7  -4⋅9, 2⋅1 | 0⋅37  0⋅43 | Ref.  0⋅52  -1⋅18 | -1⋅6, 2⋅7  -3⋅5, 1⋅2 | 0⋅63  0⋅33 |

*Based on 144 participants (2.6%) delivered by Caeserian section; 73 males and 71 females.

***Table S4***: **Change in FVC stratified by sex (model 1).** Change in FVC (ΔFVC = in ml per unit per year) from wave 1 to 2, 2 to 3 and 1 to 3, stratified on sex. The estimates are adjusted for age, height, FVC at baseline (ECRHS1 / NFBC1966 I) (model 1).
