# Supplementary material for: Early life exposures contributing to accelerated lung function decline in adulthood – a follow-up study of 11,000 adults from the general population
Source: eClinicalMedicine. 2023 Dec 8;66:102339. doi: 10.1016/j.eclinm.2023.102339 (PMC10714210; doi:10.1016/j.eclinm.2023.102339)
Supplement: Supplementary Table S5 [file mmc7.docx]

| **Early life risk factors** | **Δ FVC (in ml per unit per year)** | | | | | |
| --- | --- | --- | --- | --- | --- | --- |
|  | **Males** | | | **Females** | | |
|  | β | 95% CI | p-value | β | 95% CI | p-value |
| Mother’s age at birth  *Age ≤19 years*  *Age 20 through 24 years*  *Age 25 through 29 years*  *Age 30 through 34 years*  *Age 35 through 39 years*  *Age ≥ 40 years* | Ref.  0⋅44  -0⋅39  -1⋅53  1⋅24  -0⋅75 | -4⋅8, 5⋅7  -5⋅6, 4⋅9  -6⋅9, 3⋅8  -4⋅4, 6⋅9  -7⋅1, 5⋅7 | 0⋅70  p-value for trend  = 0⋅75 | Ref.  -2⋅05  -3⋅60  -2⋅62  -2⋅25  -3⋅63 | -5⋅5, 1⋅3  -6⋅9, -0⋅3  -6⋅0, 0⋅8  -5⋅9, 1⋅4  -7⋅7, 0⋅5 | 0⋅26  p-value  for trend  = 0⋅28 |
| Mother smoked during pregnancy  *No*  *Yes* | Ref.  1⋅82 | -1⋅9, 5⋅5 | 0⋅34 | Ref.  1⋅47 | -0⋅7, 3⋅6 | 0⋅18 |
| Father smoked during childhood  *No*  *Yes* | Ref.  1⋅49 | -1⋅2, 4⋅1 | 0⋅27 | Ref.  0⋅15 | -1⋅7, 1⋅9 | 0⋅87 |
| Caesarean section*  *No*  *Yes* | Ref.  5⋅28 | -2⋅9, 13⋅5 | 0⋅21 | Ref.  1⋅41 | -4⋅0, 6⋅9 | 0⋅61 |
| Season of birth  *Other seasons*  *Winter* | Ref.  -0⋅18 | -2⋅4, 2⋅1 | 0⋅88 | Ref.  1⋅33 | -0⋅2, 2⋅8 | 0⋅086 |
| Mother having asthma  *No*  *Yes* | Ref.  2⋅73 | -1⋅5, 6⋅9 | 0⋅20 | Ref.  0⋅69 | -1⋅7, 3⋅1 | 0⋅57 |
| Father having asthma  *No*  *Yes* | Ref.  4⋅32 | 0⋅1, 8⋅5 | 0⋅045 | Ref.  1⋅30 | -1⋅2, 3⋅8 | 0⋅31 |
| Severe respiratory infection < 5 years  *No*  *Yes* | Ref.  3⋅61 | -0⋅8, 8⋅1 | 0⋅11 | Ref.  0⋅39 | -2⋅4, 3⋅2 | 0⋅78 |
| Mother’s education level  *Minimum school leaving age*  *Secondary school*  *College or university* | Ref.  -1⋅51  0⋅02 | -3⋅9, 0⋅9  -3⋅7, 3⋅7 | 0⋅21  0⋅99 | Ref.  -1⋅26  -2⋅07 | -2⋅9, 0⋅33  -4⋅6, 0⋅48 | 0⋅12  0⋅11 |
| Father’s education level  *Minimum school leaving age*  *Secondary school*  *College or university* | Ref.  -1.41  -1.26 | -4⋅6, 1⋅8  -4⋅9, 2⋅3 | 0⋅39  0⋅49 | Ref.  0⋅28  -1⋅03 | -1⋅9, 2⋅5  -3⋅4, 1⋅4 | 0⋅81  0⋅40 |

*Based on 144 participants (2.6%) delivered by Caeserian section; 73 males and 71 females.

***Table S5:*** **Change in FEV_1_ stratified by sex and adjusted for personal smoking (model 2).** Change in FVC (ΔFVC = in ml per unit per year) from wave 1 to 2, 2-3 and 1 to 3, stratified on sex. The estimates are adjusted for age, height, FVC at baseline (ECRHS1 / NFBC1966 I) and personal smoking (pack years) (model 2).
