# Supplementary material for: Early life exposures contributing to accelerated lung function decline in adulthood – a follow-up study of 11,000 adults from the general population
Source: eClinicalMedicine. 2023 Dec 8;66:102339. doi: 10.1016/j.eclinm.2023.102339 (PMC10714210; doi:10.1016/j.eclinm.2023.102339)
Supplement: Supplementary Table S6 [file mmc8.docx]

| **Early life risk factors** | **ECRHS** | | **NFBC1966** | |
| --- | --- | --- | --- | --- |
|  | **Δ FEV_1_**  **(in ml per unit per year)** | | **Δ FEV_1_**  **(in ml per unit per year)** | |
|  | β | 95% CI | β | 95% CI |
| Mother’s age age at birth:  *Age ≤19 years*  *Age 20 through 24 years*  *Age 25 through 29 years*  *Age 30 through 34 years*  *Age 35 through 39 years*  *Age ≥ 40 years* | Ref.  -0⋅39  -1⋅54  -1⋅33  -0⋅69  -2⋅29 | -3⋅7, 2⋅9  -4⋅8, 1⋅8  -4⋅7. 2⋅0  -4⋅3, 2⋅9  -6⋅4, 1⋅8 | Ref.  -1⋅61  -1⋅54  -3⋅13  -2⋅12  -1⋅50 | -4⋅5, 1⋅3  -4⋅4, 1⋅4  -6⋅2, -0⋅07  -5⋅3, 1⋅1  -5⋅1, 2⋅1 |
| Mother smoked during pregnancy  *No*  *Yes* | Ref.  1⋅30 | -0⋅9, 3⋅5 | Ref.  3⋅25 | 1⋅2, 5⋅3 |
| Season of birth  *Other seasons*  *Winter* | Ref.  -0⋅14 | -1⋅5, 1⋅19 | Ref.  0⋅24 | -1⋅3, 1⋅8 |
| Mother having asthma  *No*  *Yes* | Ref.  3⋅38 | 0⋅9, 5⋅9 | Ref.  1⋅32 | -0⋅9, 3⋅5 |
| Father having asthma  *No*  *Yes* | Ref.  1⋅64 | -0⋅8, 4⋅1 | Ref.  1⋅28 | -1⋅1, 3⋅7 |
| Severe respiratory infection within 5 years of age  *No*  *Yes* | Ref.  1⋅86 | -0⋅08, 3⋅8 | NA |  |
| Severe respiratory infection within 1 year of age  *No*  *Yes* | NA |  | Ref.  0⋅98 | -2⋅7, 4⋅7 |
| Age at menarche  *Early (<12 years)*  *Normal (12 – 14 years)*  *Late (> 14 years)* | 1⋅94  Ref.  -0⋅31 | -0⋅2, 4⋅1  -2⋅7, 2⋅1 | 0⋅012  Ref.  0⋅91 | -2⋅3, 2⋅4  -1⋅5, 3⋅3 |
| Mother’s education level  *Minimum school leaving age*  *Secondary school*  *College or university* | Ref.  -0⋅27  0⋅45 | -1⋅8, 1⋅3  -1⋅6, 2⋅5 | Ref.  -0⋅64  0⋅18 | -2⋅1, 0⋅8  -3⋅4, 3⋅8 |

***Table S6***: **Change in FEV_1_ across the follow-ups stratified on cohort ECHRS and NFBC1966**. The estimates are adjusted for sex, age, height and FEV_1_ at baseline (model 1). A positive number implies an accelerated decline in FEV_1_ (ml per year) compared to referents, while a negative number implies a lower decline.
