# Supplementary material for: Early life exposures contributing to accelerated lung function decline in adulthood – a follow-up study of 11,000 adults from the general population
Source: eClinicalMedicine. 2023 Dec 8;66:102339. doi: 10.1016/j.eclinm.2023.102339 (PMC10714210; doi:10.1016/j.eclinm.2023.102339)
Supplement: Supplementary Table S7 [file mmc9.docx]

| **Early life risk factors** | **ECRHS** | | **NFBC1966** | |
| --- | --- | --- | --- | --- |
|  | **Δ FVC**  **(in ml per unit per year)** | | **Δ FVC**  **(in ml per unit per year)** | |
|  | β | 95% CI | β | 95% CI |
| Mother’s age at birth:  *Age ≤19 years*  *Age 20 through 24 years*  *Age 25 through 29 years*  *Age 30 through 34 years*  *Age 35 through 39 years*  *Age ≥ 40 years* | Ref.  -1⋅64  -2⋅92  -2⋅29  -1⋅37  -2⋅37 | -5⋅9, 2⋅6  -7⋅1, 1⋅2  -6⋅5, 1⋅9  -5⋅9, 3⋅1  -7⋅5, 2⋅8 | Ref.  0⋅21  -0⋅77  -2⋅06  -0⋅50  -2⋅55 | -3⋅4, 3⋅9  -4⋅4, 2⋅9  -5⋅9, 1⋅8  -4⋅5, 3⋅5  -7⋅0, 2.0 |
| Mother smoked during pregnancy  *No*  *Yes* | Ref.  1⋅78 | -1⋅0, 4⋅6 | Ref.  1⋅98 | -0⋅5, 4⋅5 |
| Season of birth  *Other seasons*  *Winter* | Ref.  0⋅52 | -1⋅2, 2⋅2 | Ref.  0⋅62 | -1⋅3, 2⋅5 |
| Mother having asthma  *No*  *Yes* | Ref.  1⋅69 | -1⋅5, 4⋅8 | Ref.  1⋅23 | -1⋅5, 4.0 |
| Father having asthma  *No*  *Yes* | Ref.  3⋅41 | 0⋅3, 6⋅5 | Ref.  1⋅28 | -1⋅1, 3⋅7 |
| Severe respiratory infection < 5 years of age  *No*  *Yes* | Ref.  2⋅02 | -0⋅4, 4⋅5 | NA |  |
| Severe respiratory infection < 1 year of age  *No*  *Yes* | NA |  | Ref.  -0⋅63 | -5⋅2, 3⋅9 |
| Age at menarche  *Early (<12 years)*  *Normal (12 – 14 years)*  *Late (> 14 years)* | 2⋅42  Ref.  -0⋅96 | -0⋅3, 5⋅1  -4.0, 2⋅1 | 1⋅27  Ref.  0⋅10 | -1⋅6, 4⋅1  -2⋅9, 3⋅1 |
| Mother’s education level  *Minimum school leaving age*  *Secondary school*  *College or university* | Ref.  -2⋅24  -1⋅95 | -4⋅2, -0⋅3  -4⋅5, 0⋅7 | Ref.  0⋅31  6⋅64 | -1⋅5, 2⋅1  2⋅2, 0⋅1 |

***Table S7***: **Change in FVC across the follow-ups stratified on cohort ECHRS and NFBC1966**. The estimates are adjusted for sex, age, height and FVC at baseline (model 1). A positive number implies an accelerated decline in FVC (ml per year) compared to referents, while a negative number implies a lower decline.
